# Post-Ictal Mania: A Case Report with Literature Revue

**DOI:** 10.1192/j.eurpsy.2024.1012

**Published:** 2024-08-27

**Authors:** W. Haouari, S. Omri, A. Labyadh, R. Feki, I. Gassara, N. Smaoui, J. Ben Thabet, M. B. Maalej, M. Maalej, N. Charfi, L. Zouari

**Affiliations:** Psychiatry C, Hedi Chaker university Hospital, Sfax, Tunisia

## Abstract

**Introduction:**

While postictal mania is a well-recognized clinical condition, it has received less research attention compared to other postictal manifestations.

**Objectives:**

Drawing upon an analysis of a case report that underscores the clinical and therapeutic challenges associated with comorbid epilepsy and mania, a literature review was carried out to investigate the connection between these two disorders.

**Methods:**

We illustrate a case of comorbidity between mania and epilepsy and provide a concise review of the literature summarizing the key characteristics of this association.

**Results:**

This case pertains to Mr. M, a 44-year-old male with a history of frontal epilepsy characterized by secondary partial generalization, which was partially controlled with sodium valproate. He was admitted to our service due to acute agitation following a loss of consciousness lasting a few minutes.

Upon admission, the patient exhibited symptoms of mental confusion. A neurological examination did not uncover any abnormalities. Brain computed tomography revealed mild frontal atrophy. Video electroencephalography conducted during the interictal period and outside the episodes of confusion did not reveal any abnormalities. The patient was restarted on sodium valproate (20 mg/kg/day) and clonazepam (2 mg/day). Following a lucid interval of ten days, the patient started to manifest psychiatric symptoms, which included irritability, hostility towards his spouse, increased talkativeness, thought pressure, and an unusual sense of familiarity, raising suspicion of post-ictal mania.

**Conclusions:**

Based on this clinical case and the existing scientific literature, post-ictal mania occupies a distinct position among the mental disorders observed in the post-ictal period. Therefore, clinicians must be aware of these conditions to facilitate accurate diagnosis and appropriate management.

**Disclosure of Interest:**

None Declared

